# *GSTP1* rs1138272 Polymorphism Affects Prostate Cancer Risk

**DOI:** 10.3390/medicina56030128

**Published:** 2020-03-13

**Authors:** Veljko Santric, Milica Djokic, Sonja Suvakov, Marija Pljesa-Ercegovac, Marina Nikitovic, Tanja Radic, Miodrag Acimovic, Vesna Stankovic, Uros Bumbasirevic, Bogomir Milojevic, Uros Babic, Zoran Dzamic, Tatjana Simic, Dejan Dragicevic, Ana Savic-Radojevic

**Affiliations:** 1 Clinic of Urology, Clinical Center of Serbia, 11000 Belgrade, Serbia; veljkosantric@yahoo.com (V.S.); miodrag.acimovic@med.bg.ac.rs (M.A.); urosbu@gmail.com (U.B.); em2bogomir@yahoo.com (B.M.); urosb2001@yahoo.com (U.B.); slavica.lisicic@kcs.ac.rs (Z.D.); 2 Institute for Oncology and Radiology of Serbia, 11000 Belgrade, Serbia; mdjokicx@gmail.com (M.D.); marina.nikitovic@ncrc.ac.rs (M.N.); vesna.stankovic@ncrc.ac.rs (V.S.); 3 Institute of Medical and Clinical Biochemistry, 11000 Belgrade, Serbia; sonja.suvakov@gmail.com (S.S.); m.pljesa.ercegovac@gmail.com (M.P.-E.); tanjajevtic@gmail.com (T.R.); tatjana.simic@med.bg.ac.rs (T.S.); 4 Faculty of Medicine, University of Belgrade, 11000 Belgrade, Serbia; 5 Serbian Academy of Sciences and Arts, 11000 Belgrade, Serbia

**Keywords:** GSTP1, polymorphism, prostate cancer, risk, haplotype

## Abstract

*Background and Objectives*: One of the most frequent genetic alterations reported to date in prostate cancer (PC) is aberrant methylation of glutathione transferase P1 (*GSTP1*). Taking into consideration the involvement of oxidative stress in PC pathogenesis and recent advances in scientific understanding of the role of GSTP1*Ala114Val rs1138272 polymorphism in carcinogenesis, we hypothesized that this single-nucleotide polymorphism (SNP) influences the risk of PC independently of, or in combination with, other GST polymorphisms, including GSTP1*IIe105Val rs1695 or *GSTM1* and *GSTT1* deletion polymorphisms. *Materials and Methods*: Genotyping was performed in 237 PC cases and in 236 age-matched controls by multiplex polymerase chain reaction (PCR) for deletion of GST polymorphisms and by quantitative PCR for SNPs. *Results*: We found that carriers of either GSTP1*Val (rs1138272) or GSTP1*Val (rs1695) variant alleles had a PC risk compared to individuals with both referent alleles (OR = 4.93, 95%CI: 2.89–8.40, *p* < 0.001 and OR = 1.8, 95%CI: 1.19–2.73, *p* = 0.006, respectively). Additionally, in a haplotype analysis we found that individuals with GSTP1*C haplotype, represented by both variant alleles (GSTP1*Val rs1695 + GSTP1*Val rs1138272), had a 5.46 times higher risk of PC development compared to individuals with the most frequent haplotype (95%CI = 2.56–11.65, *p* < 0.001), suggesting a potential role of those variants in PC susceptibility. A regression analysis on the number of risk-associated alleles per individual (GSTM1*active, GSTT1*null, GSTP1*Val rs1695 and GSTP1*Val rs1138272) showed a significant increase in the risk of developing PC, from 3.65-fold in carriers of two risk alleles (95%CI = 1.55–8.61, *p* = 0.003) to an approximately 12-fold increase in carriers of all four risk alleles (95%CI = 3.05–44.93, *p* < 0.001). *Conclusion*: Prostate cancer may be influenced by multiple glutathione transferase (GST) polymorphic genes, especially *GSTP1*, highlighting the role of gene–gene interactions in human susceptibility to this cancer.

## 1. Introduction

Prostate cancer (PC) represents the fourth most prevalent cancer and the sixth leading cause of cancer death worldwide [1]. Moreover, this is the second most frequent cancer diagnosis and the second cause of all cancer deaths among men reported in the USA and the European Union [2]. At the early stage, PC might be asymptomatic, and thus requires only active surveillance. Nevertheless, the accurate assessment of men who are more prone to developing clinically apparent PC and the further estimation of disease progression still represents a great dilemma [3]. These varying clinical responses might also be attributed to established PC risk factors, including advanced age, ethnicity, genetic factors and family history [4].

In PC carcinogenesis, one of the most crucial underlying molecular mechanisms involves a complex interplay between oxidative stress, chronic inflammation and androgen receptor (AR) mediated-signaling [5,6]. Moreover, it has been proposed that oxidative stress is not only inherent in PC development, but is critical for the development of the aggressive phenotype [5]. Numerous PC-related factors, such as aging, the antioxidant system, androgen imbalance, dietary fat and premalignant conditions, may be associated with the occurrence of oxidative stress. In response to disturbances in redox homeostasis, the consequent activation of transcription factor Nrf2 induces an expression of genes that have one or more antioxidant response elements (ARE) in their promoter regions, including members of the glutathione transferase (GST) enzyme superfamily [7]. As a part of the Phase II detoxification system, GSTs exhibit catalytic activity by the formation of thioether conjugates, which mainly result in the detoxification of a variety of small molecule electrophiles [8,9]. One of the common genetic alterations in PC reported to date is the silencing of the gene for GSTP1 isoenzyme, implying its particularly important antioxidant and detoxification role with respect to PC carcinogenesis [10]. Specifically, the methylathion of the *GSTP1* gene occurs exclusively in PC tissue, in contrast to healthy and benign prostate tissue where this gene inactivation does not take place [10]. Moreover, the presence of mGSTP1 in the circulatory system is associated with tumor aggressiveness. On the other hand, GTSP1 overexpression of prostate cancer cells inhibits cell viability and motility by targeting proto-oncogene MYC [11].

It is important to note that polymorphisms in *GSTP1* and other GSTs could also contribute to PC occurrence and progression, affecting both the proliferation capacity of tumor cells and their response to therapy [9,12]. To date, *GSTM1-null* and *GSTT1-null* genotypes have been the focus of numerous investigations attempting to elucidate the effect of their deficiency and susceptibility to cancers. The underlying hypothesis in these studies is that the homozygous deletion of the *GSTM1* and *GSTT1* genes, due to their impaired ability to detoxify electrophilic carcinogens, may increase their susceptibility to somatic DNA mutations and, therefore, place *GSTM1-null* and *GSTT1-null* individuals at increased risk of cancer [8]. The investigations which estimated the role of *GSTM1* and *GSTT1* deletion polymorphisms in PC development showed an association between the *GSTM1-null* genotype and a significant increase in PC susceptibility among Asians and Eurasians [13,14], but not European populations, as a recent meta-analysis revealed [15]. Moreover, it has been shown that the *GSTT1* deletion polymorphism may be a strong indicator of prostate cancer among Africans [15]. Regarding *GSTP1* polymorphisms, whose gene is located on chromosome 11 (11q13.2) [16], two commonly occurring polymorphisms within the exon 5/6 region of the gene (rs1695 c.313A > G, p.IIe105Val and rs1138272 c.341C > T, p.Ala114Val) may be related to the occurrence and development of various cancers [17,18,19,20,21]. Moreover, those *GSTP1* polymorphic variants generate four specific *GSTP1* haplotypes—GSTP1*A (Ile105/Ala114), GSTP1*B (Val105/Ala114), GSTP1*C (Val105/Val114) and GSTP1*D (Ile105/Val114)—whose specific association with alterations in catalytic and regulatory GSTP1 roles might have a potential clinical significance in disease susceptibility and response to oxidative stress [22]. It should be noted that *GSTP1* rs1695 polymorphism, based on several meta-analyses, is likely to be associated with a risk of prostate cancer, [23,24] while the data on the association between GSTP1*Ala114Val rs1138272 polymorphism and PC risk are scarce.

Taking into consideration the involvement of oxidative stress in PC pathogenesis and recent advances in scientific understanding of the role of GSTP1*Ala114Val rs1138272 in carcinogenesis, we hypothesized that this SNP influences the risk of PC independently of, or in combination with, another GST polymorphisms such as GSTP1*IIe105Val rs1695 or *GSTM1* and *GSTT1* deletion polymorphisms. Therefore, we performed a comprehensive analysis of *GSTM1* and *GSTT1* deletion polymorphisms, as well as of *GSTP1* rs1138272 and GSTP1*IIe105Val rs1695 polymorphisms in a cohort of 237 prostate cancer cases and 236 age-matched controls.

## 2. Materials and Methods

### 2.1. Subjects

We enrolled 237 patients (average age: 68.81 ± 6.91 years) from the Urology Clinic, Clinical Center of Serbia, Belgrade, and the Institute for Oncology and Radiology of Serbia, Belgrade, Serbia, with histologically confirmed PC by pathologists specialized in uropathology. After informed consent was obtained, each subject was interviewed using a standard questionnaire, composed at the Institute of Epidemiology, University of Belgrade Faculty of Medicine (UBFM), to collect information on demographic characteristics. Data on prostate cancer diagnostics and treatment were taken from medical records and medical history, whereas data on demographics, physical activity and reproductive and benign prostate hyperplasia history were obtained by a questionnaire. The control group, who were matched to PC patients according to age, comprised 236 individuals (average age: 67.35 ± 9.18 years) recruited from the Urology Clinic, Clinical Center of Serbia, Belgrade, with no previous personal history of malignant disease.

All participants signed a statement providing their written informed consent. The Ethical Committee of the UBFM approved the study protocol (approval number: 2650/IV-21, 10 April 2018) and the research was carried out in compliance with the Declaration of Helsinki.

### 2.2. GST Genotyping

DNA from the whole blood was isolated using a QIAamp DNA mini kit (Qiagen, Hilden, Germany). *GSTM1* and *GSTT1* genotyping was performed by multiplex PCR [25]. The primers used for *GSTM1* were forward 5′-GAACTCCCTGAAAAGCTAAAGC-3′ and reverse 5′-GTTGGGCTCAAATATACGGTGG-3′. The primers used for *GSTT1* were forward 5′-TTCCTTACTGGTCCTCACATCTC-3′ and reverse 5′-TCACGGGATCATGGCCAGCA-3′. Exon 7 of the *CYP1A1* gene, that was co-amplified using forward 5′-CAGCTGCATTTGGAAGTGCTC-3′ and reverse 5′-CAGCTGCATTTGGAAGTGCTC-3′ primers, was used as an internal control. Since the assays did not distinguish between referent homozygous and heterozygous genotypes, the presence of an active genotype was detected by a 215 bp band for *GSTM1* and a 480 bp band for *GSTT1*.

The GSTP1*IIe105Val rs1695 and GSTP1*Ala114Val rs1138272 genotypes were determined by qPCR using Applied Biosystem Taqman Drug Metabolism Genotyping assays with the identification numbers *C_3237198_20* and *C_1049615_20*, respectively (Applied Biosystems™, Foster City, CA, USA). After diluting genomic DNA to a final concentration of 6ng/µL, 5µL of the sample was applied into each well of the reaction plate and dried down at 65°C in 30 min. Next, 0.25 µL of TaqMan probe, 2.50 µL of commercial MasterMix and 2.25 µL of DNAse-free water were mixed in a total volume of 5 µL and added to the plate. The thermal protocol for gene amplification included 4 min of initial denaturation and 40 repeated cycles (15s at 95 °C and 1 min at 60 °C), after which the genotypes were analyzed according to the Eppendorf real plex software instructions.

### 2.3. Statistical Analysis

A statistical analysis was performed using SPSS (SPSS Inc., Chicago, Illinois, USA) ver.17.0. Odds ratios (ORs) and corresponding 95% confidence intervals (CIs) were computed by multi-nominal logistic regression in order to calculate the association between genotypes and the risk of prostate cancer development. The results were adjusted using age, hypertension and diabetes mellitus type 2 as confounding factors. A χ2 test was used to test the deviation of the genotype distribution using the Hardy–Weinberg equilibrium. An evaluation of the linkage disequilibrium (LD) between SNPs and the haplotype analysis was performed using the SNPStats software available online [26]. The strength of LD was expressed by D′ = D/Dmax. For all analyses, p values <0.05 were considered significant.

## 3. Results

The demographic and clinical characteristics of patients and controls are presented in Table 1. There was no statistically significant difference in age, body mass index and smoking habits (*p* > 0.05), whereas the presence of diabetes and hypertension was significantly higher in the patient group compared to the control group (*p* < 0.001 and *p* = 0.002, respectively). As presented in Table 1, prostate-specific antigen (PSA) at diagnosis >20 ng/mL was shown to be the most frequent among PC patients (36%). Regarding Gleason score, the majority of patients had GS 7 (3 + 4) (30%) (Table 1).

The distribution of the GSTs genotypes is presented in Table 2. The Hardy–Weinberg equilibrium of all GST genotypes was confirmed for both the patients and controls (*p* > 0.05). There was no statistically significant association between either the *GSTM1* or the *GSTT1* genotype and PC risk (*p* > 0.05). In contrast, carriers of at least one variant GSTP1*Val (rs1695) allele were at a 1.8-fold higher risk of developing PC compared to carriers of both referent alleles (*p* = 0.006). The increased risk was further potentiated in carriers of both variant GSTP1*Val (rs1695) alleles (OR = 1.99, 95%CI: 1.08–3.68, *p* = 0.028). Similarly, GSTP1*Val (rs1138272) allele carriers had a nearly 5-fold higher risk of cancer development compared to individulas with both referent alleles (OR = 4.93, 95%CI: 2.89–8.40, *p* < 0.001). The risk was increased to more than 7-fold in individulas with both variant GSTP1*Val (rs1138272) alleles (OR = 7.16, 95%CI: 1.54–33.26, 0.012).

Considering that GSTP1*IIe105Val rs1695 and GSTP1*Ala114Val rs1138272 are separated by only approximately 1kb, linkage disequilibrium (LD) and haplotype analyses were performed. In this LD analysis, we found a D’ of 0.48, indicating the presence of LD between the SNPs. The most frequent haplotype was GSTP1*A in patients (51%), as well as in controls (64%), consisting of referent alleles in both *GSTP1* SNPs (GSTP1*Ile rs1695 + GSTP1*Ala rs1138272) (Table 3). Carriers of the GSTP1*C haplotype, represented by both variant alleles (GSTP1*Val rs1695 + GSTP1*Val rs1138272), had a 5.46-times higher risk of development of prostate cancer compared to individuals with the most frequent haplotype (OR = 5.46, 95%CI = 2.56–11.65, *p* < 0.001) (Table 3). The least prevalent haplotype was GSTP1*D in patients (8%) and in controls (2%)*,* consisting of a referent allele in GSTP1 rs1695 (GSTP1*Ile) and variant *GSTP1 rs1138272* allele (GSTP1*Val). Nevertheless, individuals with this haplotype were at an approximately 2.5–fold higher risk of developing PC when compared to the GSTP1*A haplotype (OR = 2.40, 95%CI = 1.08-5.34, *p* = 0.033) (Table 3).

To evaluate the potential cumulative effect of these GST polymorphisms on susceptibility to prostate cancer development, a regression analysis was performed based on the number of risk-associated alleles per individual (GSTM1*active, GSTT1*null, GSTP1*Val rs1695 and GSTP1*Val rs1138272). The results, which are demonstrated in Table 4, showed a statistically significant increase in the risk of developing PC, from 3.65–fold in carriers of two risk alleles (OR = 3.65, 95%CI = 1.55-8.61, *p* = 0.003) to an approximately 12–fold increase in carriers of all four risk alleles (OR = 11.71, 95%CI = 3.05–44.93, *p* < 0.001).

## 4. Discussion

The results of this study have shown that men with at least one copy of the variant *GSTP1* allele rs1138272 (*Ala/Val + Val/Val) or *GSTP1* rs1695 (*Ile/Val + Val/Val) are at a significantly higher risk of prostate cancer. The effect on PC susceptibility was even more pronounced when both *GSTP1* variant alleles were present in combination. Indeed, a haplotype analysis has shown that carriers of the GSTP1*C haplotype, represented by both *GSTP1* variant alleles, has borne an almost five-times higher risk of PC in comparison to carriers of GSTP1*A (both wild-type alleles). On the other hand, polymorphic expression of *GSTM1* or *GSTT1* was not independently associated with the risk of PC, but, however, exhibited an additive effect in individuals with variant *GSTP1* alleles.

Functional polymorphisms of the GST family have the capacity to influence individual responses to environmental stresses, including oxidative stress. Extensive research has been carried out to study the relationship between borne rs1695 and PC susceptibility, including several meta-analyses with conflicting conclusions [23,24,27,28]. Thus, the study by Cai et al. found a significant association among Caucasians in ethnicity-based subgroup analyses, contrary to the findings on Asians and African Americans [28]. The results of another meta-analysis showed a significant association between GSTP1*Ile105Val polymorphism and PC risk, but only among Asians [23]. Nevertheless, a comprehensive meta-analysis conducted on a study group comprising 5301 cases and 5621 controls found no association between GSTP1*Ile105Val polymorphism and the risk of PC [27]. The results of our study are in favor of the previously observed role of GSTP1*Ile105Val polymorphism in prostate carcinogenesis, since increased PC risk was found in individuals bearing at least one variant GSTP1*Val allele.

The significance of another GSTP1*Ala114Val rs1138272 polymorphism in cancer susceptibility has emerged recently. It has been shown that the GSTP1*T/T genotype is likely to be related to overall cancer susceptibility among the Asian and African population and specifically, colorectal and head and neck cancers in the Caucasian population. In addition, the GSTP1*CT genotype may be linked to the risk of lung cancer in Caucasians [29,30,31]. In contrast to GSTP1*Ile105Val rs1695 polymorphism, the modifying effect of GSTP1*Ala114Val rs1138272 polymorphism on the risk of prostate cancer has been investigated in only two studies [32,33]. These studies found that among SNPs implicated in the steroid pathway, the GSTP1*Ala114Val rs1138272 polymorphism was associated with prostate volume in Caucasian men with localized prostate cancer treated by radical prostatectomy [32].

It seems that the functional relevance of both *GSTP1* polymorphisms in PC susceptibility might be related to changes in both the catalytic and regulatory functions of GSTP1. The genetic variation posed by Ile to Val substitution at the 105 site results in the steric restriction of the H-site, which might affect substrate specificity that differs from those of the wild-type allozyme. Thus, the GSTP1*105Val variant allozyme may be able to accommodate less bulky substrates than the GSTP1*105Ile allozyme [22,34]. In case of GSTP1*Ala114Val rs1138272 polymorphism, changes in substrate specificity are due to an altered ability to distinguish between planar and non-planar substrates [22]. Despite the fact that GSTP1 is involved in the detoxification of epoxides from carcinogenic polycyclic aromatic hydrocarbons present in cigarette smoke, the results of a recent study did not support the proposition that smoking modifies the effect of GSTP1*Ile105Val polymorphism on prostate cancer risk [35]. Another group of possibly carcinogenic GSTP1 substrates are pesticides, which have been increasingly suggested as work-related PC risk factors [36,37]. Last, but not least, exposure to heavy metals which interact with GSTP1, especially cadmium and mercury, has been shown to play a role in PC [36,38,39]. Thus, in an experimental model of prostate carcinogenesis induced by cadmium, a lower GSTP1 expression influenced the response to oxidative stress in the dysplastic changes caused by cadmium [40]. In this line of research, very recently, Chang et al. have shown that levels of cadmium (Cd), nickel (Ni), and copper (Cu) were significantly higher in patients with prostatic hyperplasia than in controls, while mercury (Hg) levels were highest in PC patients [41]. Interestingly, epidemiological studies found associations between GST polymorphisms, including GSTP1*Ile105Val and GSTP1*Ala114Val, and interindividual differences in metabolism and the elimination of Hg and arsenic [42,43,44]. Limited epidemiological studies, as well as in vitro results, suggest that GSTP1*Ile105Val and GSTP1*Ala114Val polymorphisms might have the potential to influence Hg toxicokinetics. Indeed, GSTP1 variants exhibit differential enzymatic activity and inhibition by heavy metals, including Hg [45]. Since the relationship between GSTP1 polymorphisms, Hg exposure and the risk of prostate cancer seems biologically plausible, it merits future studies in a larger population.

In addition to the classic catalytic functions, the GSTP1 enzyme also exhibits regulatory roles that impact cell survival pathways, specifically the mitogen-activated protein kinase (MAPK) [46]. Moreover, different GSTP1 polymorphic variants, with differences in residues 105 and 114, triggered different regulatory effects. Thus, it was verified that the GSTP1*C haplotype is a more potent inhibitor of MAPK-C-Jun N-terminal kinase (JNK) activity than the wild-type GSTP1*A [47]. Keeping in mind JNK’s roles in the apoptosis, proliferation, migration and DNA repair in prostate cancer, as well as the relationship between JNK and the androgen receptor, it might be hypothesized that GSTP1 polymorphic variants might have different impacts on PC development [48]. To our knowledge, this is the first report on the association of the GSTP1*C haplotype with a risk of prostate cancer. Given the important functions of GSTP1, it seems reasonable to suggest that *GSTP1* polymorphisms may modify the risk of localized prostate cancer. Since the downregulation of GSTP1 expression is a hallmark of prostate cancer, it may be speculated that polymorphic expression influences tumor development in the early stages of prostate carcinogenesis, due to quantitative and qualitative differences in GSTP1 expression and function, respectively. In later stages, methylation of the *GSTP1* gene overwhelms the effect of the variant genotype, since the silencing of GSTP1 affects both gene variants and results in decreased protein expression and a lack of function.

Regarding a possible gene–environment interaction, it is important to note that, in our cohort of PC patients, the presence of diabetes and hypertension was significantly higher in comparison to that of the controls. It has been suggested that these confounding factors might influence aggressive and metastatic PC, as well as have some impact on the outcome after different therapeutic treatments, including androgen-deprivation therapy [3,49]. Although data on modifying the effect of GST genetic variations at risk to diabetes and hypertension are inconsistent [8,50], we made an adjustment in order to exclude the potential bias related to these confounding factors. Analyses of polymorphic GST allelic variants (polygenic mechanisms) suggest that the combined effects of both deletions and SNPs account for the overall susceptibility of individuals to xenobiotics [51]. In our study, neither *GSTM1* nor *GSTT1* deletion polymorphisms were independently associated with PC risk, however they exhibited an additive effect in individuals with variant *GSTP1* SNPs. In conclusion, prostate cancer may be influenced by multiple polymorphic genes, highlighting the role of both gene–gene and gene–environment interactions in the susceptibility of individuals to this cancer.

## 5. Conclusions

In conclusion, prostate cancer may be influenced by multiple polymorphic genes, highlighting the role of both gene–gene and gene–environment interactions in the susceptibility of individuals to this cancer. As such, our results suggest modifying the role of multiple GST polymorphic genes, especially *GSTP1*, to reduce prostate cancer risk.

## Figures and Tables

**Table 1:** Demographic and clinical characteristics of patients with prostate cancer and of controls.

	Patients, *n (%)*	Controls, *n (%)*	*p*
Age *	68.81 ± 6.91	67.35 ± 9.18	0.052
BMI *	26.98 ± 3.48	26.52 ± 3.71	0.203
Hypertension (Y/N)	125 (58)/92 (42)	87 (39)/135 (61)	<0.001
Diabetes mellitus type 2 (Y/N)	37 (16)/188 (84)	12 (7)/174 (93)	0.002
Smoking (Y/N)	111 (48)/119 (52)	104 (46)/124 (54)	0.570
Prostate-specific antigen (PSA) at diagnosis (ng/mL)			
<10	78 (34)		
10–20	69 (30)	/	/
>20	82 (36)	/	/
PSA at diagnosis * (ng/mL)	23.41 ± 27.48	/	/
Gleason score#			
≤ 6	56 (27)	/	/
7 (3 + 4)	62 (30)	/	/
7 (4 + 3)	38 (18)	/	/
8	28 (14)	/	/
9/10	22 (11)	/	/

* mean value ± standard deviation.

**Table 2:** Distribution of GSTM1, GSTT1, GSTP1 rs1695 and GSTP1 rs113272 polymorphisms in patients with prostate cancer and in controls.

Genotype	Patients, *n (%)*	Controls, *n (%)*	OR (95% CI) ^a^	*p*
*GSTM1*				
*GSTM1-active*	147 (62)	131 (56)	1.0	
*GSTM1-null*	90 (38)	104 (44)	0.77 (0.51–1.15)	0.203
*GSTT1*				
*GSTT1-active*	148 (62)	156 (66)	1.0	
*GSTT1*-*null*	89 (38)	79 (34)	1.11 (0.73–1.70)	0.625
*GSTM1/GSTT1*				
*M1/T1-active*	86 (36)	85 (36)	1.0	
*M1-null/T1-active*	62 (26)	71 (30)	0.85 (0.51–1.41)	0.525
*M1-active/T1-null*	61 (26)	46 (20)	1.23 (0.71–2.13)	0.453
*M1/T1-null*	28 (12)	33 (14)	0.79 (0.41–1.49)	0.461
*GSTP1 rs1695*				
*IleIle	83 (35)	107 (46)	1.0	
*IleVal	114 (48)	95 (40)	1.74 (1.12–2.72)	0.014
*ValVal	40 (17)	32 (14)	1.99 (1.08-3.68)	0.028
*IleVal + ValVal	154 (65)	127 (54)	1.80 (1.19–2.73)	0.006
*GSTP1 rs1138272*				
*AlaAla	135 (57)	184 (87)	1.0	
*AlaVal	89 (38)	26 (12)	4.71 (2.70–8.20)	<0.001
*ValVal	11 (5)	2 (1)	7.16 (1.54–33.26)	0.012
*AlaVal + ValVal	100 (43)	28 (13)	4.93 (2.89–8.40)	<0.001

^a^ OR—Odds ratio, CI 95%-95% confidence interval; values are adjusted for age, HTA and DM2T.

**Table 3:** *GSTP1 rs1695/rs1138272* haplotype distribution in prostate cancer patients and controls.

Haplotype	GSTP1 rs1695	GSTP1 rs1138272	Controls (%)	Patients (%)	OR (95% CI) ^a^	*p*
A	*A	*C	64	51	1.00	
B	*G	*C	29	25	1.33 (0.89–1.99)	0.170
C	*G	*T	5	16	5.46 (2.56–11.65)	<0.001
D	*A	*T	2	8	2.40 (1.08–5.34)	0.033

^a^ OR—Odds ratio, CI 95%-95% confidence interval; values are adjusted for age, HTA and DM type II; *p* < 0.050 was considered signifcant.

**Table 4:** Cumulative effect of number of risk-associated GST gene variants in patients with prostate cancer and in controls.

Genotype	Patients, *n* (%)	Controls, *n* (%)	OR (95%CI) ^a^	*p*
GSTM1-active, GSTT1-null, GSTP1*Val rs1695 and GSTP1*Val rs1138272				
0 risk alleles	11 (5)	25 (12)	1.0	
1 risk allele	63 (27)	81 (39)	2.06 (0.87–4.89)	0.100
2 risk alleles	82 (35)	66 (31)	3.65 (1.55–8.61)	0.003
3 risk alleles	57 (24)	34 (16)	4.30 (1.74–10.59)	0.002
4 risk alleles	22 (9)	4 (2)	11.71 (3.05–44.93)	<0.001

^a^ OR—Odds ratio, 95%CI —95% confidence interval; values are adjusted for age, HTA and DM type II; *p* < 0.050 was considered significant.
